# Establishment of a collaborative research ethics training program to prepare the next generation of ethics researchers in Mali

**DOI:** 10.1007/s40889-023-00170-0

**Published:** 2023-05-26

**Authors:** Seydou Doumbia, Heather E Rosen, Nino Paichadze, Housseini Dolo, Djeneba Dabitao, Zana Lamissa Sanogo, Karim Traore, Bassirou Diarra, Yeya dit Sadio Sarro, Awa Keita, Seydou Samake, Cheick Oumar Tangara, Hamadoun Sangho, Samba Ibrahim Diop, Mahamadou Diakite, Adnan A Hyder, Paul Ndebele

**Affiliations:** 1 Faculty of Medicine and Odonto-Stomatology, University of Sciences, Techniques and Technologies of Bamako, Bamako, Mali; 2 Present Address: University Clinical Research Center (UCRC), University of Sciences, Techniques and Technologies of Bamako, Bamako, Mali; 3 Center on Commercial Determinants of Health, Department of Global Health, Milken Institute School of Public Health, George Washington University, Washington, DC USA; 4 University Clinical Research Center (UCRC), University of Sciences, Techniques and Technologies of Bamako, Bamako, Mali; 5 Faculty of Pharmacy, University of Sciences, Techniques and Technologies of Bamako, Bamako, Mali

**Keywords:** Graduate education, Mali, Research ethics, Capacity building

## Abstract

Background: Despite an increase in health research conducted in Africa, there are still inadequate human resources with research ethics training and lack of local long-term training opportunities in research ethics. A research ethics training program named United States-Mali Research Ethics Training Program (US-Mali RETP) was established through a partnership between the George Washington University Milken Institute School of Public Health (GWSPH), USA and University of Sciences, Techniques & Technologies of Bamako (USTTB), to address the critical need for improved bioethics training, leadership, and policy in Mali. Methods: The aims of the capacity building programme are achieved by leveraging US and Africa-based expertise to strengthen research ethics education and capacity through: (1) intensive faculty development to design ethics training curricula; (2) development of a research ethics specialization within the existing Masters of Public Health program (MPH); and (3) establishing professional development courses through short-term training workshops and webinars to address research and professional needs in ethics. Results: The program will strengthen USTTB’s capacity of research ethics by training at least 15 MPH students over 5 years to prepare the next generation of ethics researchers. During the first two years of the program, a new ethics training curricula was developed, two cohorts of Master’s students were enrolled, and a series of webinars and workshops were conducted with participation of Malian and international researchers. Conclusions: US-Mali RETP will promote a sustainable bioethics enterprise at USTTB, and enable dissemination of research ethics training to increase health research capacity in Mali.

## Introduction

The volume of health research in low- and middle-income countries (LMIC) has increased exponentially in the past decades with an estimated global health research enterprise reaching over $160 billion annually (IEG-WBG 2009). Mali is one of the largest West African countries with highest burdens of both communicable and non-communicable diseases (NCDs) and has weak healthcare delivery system as well as public health research capacity despite a national policy to strengthen health research (Cilingurtuk 2018; Landoure 2016; WHO 2008). Mali has experienced a remarkable increase in health research over the past two decades, including clinical trials and research in the social, behavioral and implementation sciences (Traore 2015; Koita 2016; Diarra 2016; Shaffer 2019; Doumbia et al. 2020). Since 2002, Mali has hosted one of the US National Institute of Allergy and Infectious Diseases (NIAID) International Centers of Excellence in Research (ICER) on infectious diseases research which has contributed to building an outstanding research infrastructure and a pool of independent researchers capable of leveraging international research funding (Doumbia et al. 2020). Mali is also a site for the Target Malaria Project which is focused on gene-drive technology to create transgenic mosquitos for controlling malaria transmission in the field, a project which has ethical implications (Roberts and Thizy 2022). As a result, Mali is one of the West African countries with the highest numbers of biomedical publications between 2005 and 2014 (Nagwu 2016).

As public health research agenda expands, new health research challenges emerge, including ethics and regulatory issues requiring adequate infrastructure and trained human resources (Diarra 2016; Koita 2016). Increase in genomics research and development of new drugs and vaccines for the prevention and control of emerging communicable diseases and NCDs highlights the importance of capacity building in ethics, regulations and standards (Landoure 2016; Traore 2015; H3Africa 2014; Diarra 2016; Koita 2016).

In 2009, the Government of Mali (GOM) made ethical clearance mandatory for all health research involving human participants in the country (Law 09–59, 28 Dec 2009). The law envisions the creation of a “*health research system to prioritize, coordinate, and facilitate effective and ethical health research and its translation into products, policies and programs aimed at improving the health of Malians*” (GOM 2009). The law explicitly enshrines ethics of research as one of its central tenets; however, little systematic capacity development work has been done thus far to achieve this goal. The response of the public sector to the growth in health research has involved strong policy support as evidenced by the existence of above mentioned government regulations. Through the collaboration with the US National Institutes of Health (NIH), since 1994, Mali has established a research ethics committee (REC) at the Faculty of Medicine, Pharmacy and Odontostomatology (FMPOS) of the USTTB, one of its kind in the subregion with a Federal wide Assurance (FWA00001769). Two other institutional research ethics committees (RECs) have been established by the National Institute of Public Health (Institut National de Santé Publique (INSP)) and the Center for Sickle Cell Disease Research and Control (le Comité d’Ethique de Centre de Recherche et Lutte Contre la Drépanocytose) (CRLD)). Additionally and importantly, a National Ethics Committee for Health & Life Sciences was created housed at the Ministry of Health to review national health research (GOM 2011).

The Malian health research enterprise requires the strengthening of support systems including human resources, financial management, regulatory policies, and ethical review processes for human research. Two major gaps are a lack of trained human resources in research ethics, and a scarcity of long-term ethics training programs (Ateudjieu 2010). An informal scan of the academic institutions has revealed that Mali has only one full Professor in ethics. Short-term initiatives such as those provided through European Developing Countries Clinical Trials partnership (EDCTP), Wellcome Trust, and the NIH have been more common. For example, the development of ethics capacity through workshops, and the development of a bilateral program between the NIH and the USTTB which has offered some short term ethics training for clinicians, researchers and editors; other partners such as the Wellcome Trust, EDCTP and the World Health Organization (WHO) have also offered some short-term training courses in research ethics. All of these have been short-term efforts, associated with specific health research programs, and none have provided a dedicated ethics training program or granted a degree in research ethics. During the past two decades, various research ethics training programs have been established across Africa. The majority of these programs cater for trainees from English speaking countries and none was specifically focused on Mali and the neighbouring countries (Ndebele et al. 2014).

A scan of the Malian research landscape confirmed that there are a limited number of professionals who have the expertise to be effective members of Research Ethics Committees (RECs); in fact, of the four registered RECs in Mali, only two provide research ethics training for its members and staff, often do not have standard operating procedures, and have inadequate representation of stakeholders (Effa 2007; Martellet 2015). This scarcity of institutional training programs in bioethics, especially in the face of emerging health threats in Mali, is a serious impediment to health research. This structural challenge does not allow the country to adequately address research ethics issues where they occur, and poses a barrier to defining local risks, protecting vulnerable groups, and regulating health research. Indeed, the literature draws attention to the system-wide implication of there being “no independent academic courses in research ethics” in the country (Maiga 2011). Studies conducted in Mali to date have identified the need for improving the existing research ethics governance system to ensure that it is responsive to the growth in international collaborative research (Effa 2007; Ateudjieu 2010). Available commentaries also confirm that relatively few IRBs in Mali are properly constituted and function optimally; and training on registration, transparency, regulation, accreditation, and audits are needed (Effa 2007; Ateudjieu 2010). The USTTB is one of the main players in health research in Mali and has conducted numerous studies on clinical medicine, demographic and population issues pertaining to public health and pathobiology. The USTTB’s research development plan highlights the training of researchers to have adequate knowledge and skills in research ethics for conducting high quality clinical and biomedical research studies. Therefore, developing a program to meet these needs will contribute to improve the overall scientific integrity in Mali. In recent years, the USTTB has been collaborating with GOM and international partners to address operational ethics issues; one of the initiatives has been to strengthen research capacity through two workshops for researchers. These workshops clearly demonstrated the interest in research ethics among researchers and research staff in Mali.

In view of the need demonstrated above, we therefore proposed to address these barriers through a collaborative partnership between the George Washington University Milken Institute School of Public Health (GWSPH), USA and University of Sciences, Techniques & Technologies of Bamako (USTTB), Mali to strengthen research ethics education and ethics research in Mali. In this paper we will share our framework for this training and experience from the first two years in developing and implementing this innovative training program to enhance research ethics capacity in Mali. We hope this will initiate a larger dialogue on models for such research ethics training and be useful to institutions in Africa and other LMICs as they consider similar efforts.

## Research ethics training program

The United States-Mali Research Ethics Training Program (US-Mali RETP) builds on the existing work between GWSPH and USTTB, and on years of previous work in developing research ethics capacity in Africa by the investigators. The purpose of the previous and existing collaborations between GWSPH and USTTB have been to facilitate programs of research and education in the field of public health and medicine; and this new program provides an opportunity for the two institutions to broaden their engagement in ethics. We envisioned a program that would build human resources for ethics in Mali, develop research ethics focused on Mali, promote research on ethics in Mali, help establish teaching programs/courses on research ethics, support building REC capacity and infrastructure in Mali and create international linkages by bringing Malian ethics experts to several international consortia on ethics. The overall goal of the US-Mali RETP is *to strengthen research ethics education and research in Mali through an innovative model of sustainable capacity development to prepare the next generation of ethics researchers*. The outcome of the US-Mali RETP will be to transform the research ethics landscape in an increasingly research-intensive country in West Africa.

Our approach is based on close collaboration between GWSPH and USTTB, aimed at supporting USTTB’s vision of becoming a regional focus for research ethics. Our program builds on the research environment built at USTTB over 25 years of collaboration with the NIH. Our model focuses on using US and Africa-based expertise to strengthen USTTB’s capacity to develop and lead a new specialization in research ethics within the existing MPH program, promote a sustainable bioethics enterprise at USTTB, and enable dissemination of research ethics training to influence capacity in Mali and the West Africa region. The program is conducted in both French and English as per the desire of USTTB to develop a bi-lingual academic program. The sections below provide some details on the planned content of the training program.

### Long-term training

To enhance the pedagogical and curricular strengths of key USTTB faculty to deliver research ethics courses and mentoring in Mali, the program focuses on enhancing analytical capacity in ethics research and training for selected Mali faculty, co-development of new ethics research training curricula for USTTB, and provision of instructional and learning support to USTTB. Selected Malian faculty with doctoral degrees in various disciplines including public health, molecular immunology, human genetics, microbiology, and social sciences participated in these activities including visits to GWSPH during year 1 and Year 2. They took intensive core courses on ethics, participated in webinars on hot topic in ethic research, worked with GWSPH faculty in developing curricula for USTTB, co-delivered ethics curricula during years 2–3 of the training program, and technical support throughout the grant period. Africa-based faculty with research ethics training and expertise (ethics consultants from research ethic training progams based in other institutions and countries in Africa) also support the design of the curriculum, mentoring, and co-teaching in Bamako and via distance learning.

The RETP provides funding for 5 trainees annually for three years and aims to train a minimum of 15 trainees at Master’s level. In addition to taking the specialization-specific ethics courses, trainees are required to participate in a practicum and conduct a research project related to research ethics. For their practicum experience, the trainees can spend some time in an IRB or within a research project addressing ethical issues. In keeping with the national research priorities of Mali, trainee projects will focus mainly on the ethics of infectious disease research; ethical analysis of genomics studies; and ethics of research on NCD risks in the country. These studies will contribute to the small amount of literature which exists from and about Mali (Diallo 2005; Marshall 2010; Yoder 2002; Martellet 2015; Hurley 2018; Tounkara 2009). Our program trainees are assigned mentors from USTTB and GWSPH to enrich their training experience and support local and international networking and professional development. Table 1 provides examples of training methods that are used in ensuring a high calibre of trainees.

**Table 1:** Examples of training methods

Method	Key goal	Format	Duration
Courses	Expand research ethics knowledge and develop critical skills	Lectures, case studies, discussions, student presentations	120 academic credits over 4 semesters
Seminars	Exposure to methods and analytical approaches of research studies in research ethics	Lectures, discussions	One hour sessions monthly over 2 semesters
Tutorials	Experience practical aspects of research ethics	Lectures, discussions	90 min sessions monthly over 2 semesters
Summer Intensive Courses	Receive advanced training, provide mentoring, and expand networks	Lectures, case studies, discussions, student presentations, one-on-one sessions, site visits	One month
Thesis Research	Practical experience of planning, implementing, and evaluating a research study	One-on-one sessions	2 semesters
Webinars	Stimulate discussion on current events	Lectures, discussions	One hour sessions, occasionally
Workshops	Introduce principals and methods of research ethics	Lectures, discussions	Three days once a year

These professionals are being trained not only in general bioethics but also in ethical issues in research, responsible conduct of research, and the legal & social implications of research. The trainees develop leadership skills as they will assume senior roles in research ethics in the academic sector in Mali and serve as mentors for future leaders. By the end of five years, we anticipate that the graduates of the long-term training program will be able to conduct research and teach courses without the assistance of GWSPH faculty. They will also have supportive local and regional colleagues to provide mentorship, collaboration and motivation. The success of our trainees, however, will depend on having a network of national and regional partners committed to addressing ethics issues. We will therefore create strong linkages with other African and US programs and with the GOM (Ministries of Health and Science & Technology) and the National Ethics Committee for Health & Life Sciences.

### Short-term training and evaluation

We also provide short-term training activities at USTTB which includes workshops, symposia and a diplome course; and seminars and online webinars on key priority topics in research ethics (Table 1). Each year over 20–35 scientists and health professionals attend supplemental workshops we offer, and this will result in a critical mass of over 100–175 professionals sensitized to and trained in research ethics in Mali. The Diplome option targets individuals such as IRB members who cannot commit to fulltime study. Such individuals can take a few modules and officially receive a university qualification. The Diplome/certificate option is very popular in French speaking African countries and it is helping to train numerous scientist in a short space of time.

To ensure sustainability of research ethics capacity in Mali, our program hopes to help establish a center or unit on research within USTTB. Such a future unit will provide a home for faculty across USTTB; strengthen research ethics teaching and research capacity; enable stronger dissemination and communication; and support the REC at USTTB.

We will conduct evaluation at the individual and program levels. Individual level evaluations will focus on how the trainee met his/her own objectives and the degree to which the trainee gained new knowledge, skills, conceptual capacity, and recognition in research ethics. Program level evaluations will include a process-level evaluation (whereby trainees comment on the training program itself) and, outcome-level evaluation, where we will measure the accomplishments, development, publications, and recognition in research ethics. We will supplement objective indicators with qualitative assessment of our trainees, their levels of satisfaction and their reports of how the program did or did not fulfill their needs.

## Early progress

Table 2 presents a summary of our program achievements. Our major accomplishment during year 1 was establishing a research ethics specialization within the existing USTTB MPH program in order to train a core group of professionals with expertise in research ethics in Mali. Work under this initiative focused on developing a new curriculum in research ethics within the MPH program. The following seven courses that are worth three credits each were created as a result of the program: Bioethics, Culture, Religion & Health; Research Ethics: Issues & Analysis; Ethics of Research Methods; Ethics of Public Health and Policy; Ethics, Genetics and Genomics; Ethical Issues in International Research; and Responsible Conduct of Research. The course syllabi were developed jointly by USTTB and GWSPH faculty working collaboratively over email and video calls. Together, these courses form a comprehensive research ethics training program. Development of the new courses was initiated at the beginning of the Summer of year 1. We had planned to host members of the USTTB faculty at GWSPH in Washington, DC for a 1-month visit. Due to COVID-19, this in-person visit was converted to a virtual engagement. In order to both develop their capacity and help prepare future courses, six USTTB faculty were enrolled in two virtual week-long ethics courses held as part of the GWSPH Summer Institute.

**Table 2:** Achievements in Year 1 and Year 2 of the program

Achievement	Significance
Specialization approved at USTTB	Institutional approval of “Research Ethics” courses
Seven new course syllabi developed	Specialization curriculum established
USTTB faculty trained at GWSPH Summer Institute	Development of research ethics teaching capacity at USTTB
Recruitment of 5 trainees in Year 2	Establishment of pool of Bioethics experts in Mali
Sponsored online webinars at GWSPH	Building expertise on key priority areas
Launch of online webinars at USTTB	Building expertise on key priority areas
Three-day workshop completed at USTTB	Development of research ethics capacity at USTTB

Recruitment of the first class of trainees occurred later in the Summer 2020. From the pool of qualified applicants, we identified those with clearly stated career goals that were consistent with the anticipated training. Successful applicants have a strong academic record and relevant academic preparation, as well as impressive references that state the potential for success and commitment to a career in research ethics. Five trainees were selected, four males and one female. The trainees have strong ties with USTTB, with four of the trainees working at the University Clinical Research Center (UCRC) at USTTB. In addition, three of the trainees received medical degrees from USTTB. They are from different academic backgrounds including medical, law and social sciences (anthropology and education).

In order to promote discussion on key priority topics in research ethics, the program became a co-sponsor of a series of webinars organized by the Bioethics Interest Group (BIG) at GWSPH. The theme of these webinars was “Ethics and COVID-19” and the program sponsored 12 webinars in year 1; the webinars were open to participants from within, and outside the US-Mali RETP. In addition, USTTB hosted three of its own webinars on relevant ethics topics.

Late in 2021 the program supported a three-day workshop on Responsible Conduct of Research attended by 20 USTTB faculty and staff. The workshop served to raise awareness among health and other professionals at USTTB and Mali about the importance of research ethics. One of our program faculty member participated as facilitator in a workshop organized by the Moroccan Association of Research Ethics (MARE) and facilitated sessions on research misconduct and data management in research. This allowed for the first of many regional interactions that will foster cross-institutional learning. At the end of the 5-year program, a conference will be held to bring together all trainee cohorts and faculty to share experiences as well as findings from their respective areas of research.

## Discussion

The establishment of the US-Mali RETP will serve West Africa, Mali and USTTB in various ways. The USTTB will become an important player in research ethics in West Africa. The program will establish a pool of individuals trained in research ethics at master’s level who will also serve as trainers; and some of them will go on to do their PhD in ethics and bioethics. The program will provide short-term training opportunities for researchers, IRB members and other stakeholders. An important contribution will be the development of educational materials as well as publications on research ethics topics that shall be generated by the trainees as they will be conducting their research projects. It is hoped that the program will positively impact on the quality of ethical review by IRBs in Mali, leading to improved efficiency.

The program expects to confront challenges such as finding good candidates in an environment where ethics is almost unknown by local communities including members of other research disciplines (apart from those working in the biomedical research sector) and future employment of trainees. To address these challenges, a plan has been developed to collaborate with other universities in Mali with the goal of having diversity in candidates who come from multiple disciplines throughout the country.

We are implementing a capacity development model for Malians by Malians with technical assistance from the US to address a major gap in the health research enterprise. The US-RETP hopes to make a meaningful contribution in Mali and in West Africa by facilitating networking and dissemination of information on research ethics. In the end, however, the long-term success of the program will be reflected in the number of trainees, in an increase in the body of research ethics knowledge that supports the implementation of national and regional research policy, and the capacity to support future sustainable ethical regulations.
